# Retraction Note: Insomnia, sleepiness, anxiety and depression among different types of gamers in African countries

**DOI:** 10.1038/s41598-020-66798-w

**Published:** 2020-06-04

**Authors:** F. A. Etindele Sosso, D. J. Kuss, C. Vandelanotte, J. L. Jasso-Medrano, M. E. Husain, G. Curcio, D. Papadopoulos, A. Aseem, P. Bhati, F. Lopez-Rosales, J. Ramon Becerra, G. D’Aurizio, H. Mansouri, T. Khoury, M. Campbell, A. J. Toth

**Affiliations:** 1 Center for Advanced Studies in Sleep Medicine, Hopital du Sacré-Coeur de Montreal, Research Center of Cognitive Neurosciences, Institut Santé et Société, Université du Québecà Montreal, Québec, Canada; 2 School of Social Sciences, Department of Psychology, International Gaming Research Unit and the Cyberpsychology Group, Nottingham Trent University, Nottingham, UK; 3 School of Health, Medical and Applied Sciences, Physical Activity Research Group, Central Queensland University, Rockhampton, Australia; 4 Center for Research in Nutrition and Public Health, Autonomous University of Nuevo Leon, Monterrey, N.L. Mexico; 5 Centre for Physiotherapy and Rehabilitation Sciences, Jamia Millia Islamia, New Delhi, India; 6 Department of Biotechnological and Applied Clinical Sciences, University of L’Aquila, L’Aquila, Italy; 7 Department of Pulmonology, Army Share Fund Hospital, Athens, Greece; 8 Faculty of Medicine, Université de Montréal, Montréal, Québec Canada; 9 Department of Biomedical sciences, Faculty of Arts and Sciences, University of Montréal, Montréal, Québec Canada; 10 Department of Physical Education and Sport Sciences, University of Limerick, Limerick, Ireland; 11 Innovation and Evaluation in Health Psychology, Faculty of Psychology, Autonomous University of Nuevo Leon, Monterrey, Nuevo Leon Mexico; 12 Lero Irish Software Research Centre, University of Limerick, Limerick, Ireland

Retraction of: *Scientific Reports* 10.1038/s41598-020-58462-0, published online 06 February 2020

The Editors have retracted this Article.

After publication serious concerns were raised that the results described in the Article, in particular the outcomes of statistical analyses, are internally inconsistent and that some results are mathematically impossible. Independent post-publication peer review has confirmed that there are numerous anomalies in the data which undermine the results and conclusions.

F. A. Etindele Sosso, D. J. Kuss, C. Vandelanotte, J. L. Jasso-Medrano, M. E. Husain, G. Curcio, D. Papadopoulos, A. Aseem, P. Bhati, G. D’Aurizio, H. Mansouri, T. Khoury, M. Campbell and A. J. Toth agree with the retraction. F. Lopez-Rosales and J. Ramon Becerra have not responded to any correspondence from the Editors about this retraction.

